# Older Adults as Teaching Allies: Opportunities for Age-Friendly University Innovation

**DOI:** 10.1093/geroni/igaa057.1784

**Published:** 2020-12-16

**Authors:** Joann Montepare, Kimberly Farah

## Abstract

Changing age demographics are reshaping societies and challenging institutions of higher education to consider how they can respond to aging populations through new approaches to teaching, research, and community engagement. As well, institutions are facing a range of challenges as they look to respond to the contemporary needs of traditional-aged students. The pioneering Age-Friendly University (AFU) initiative, endorsed by GSA’s Academy for Gerontology in Higher Education (AGHE), offers a framework within which institutions can begin to address these issues through more age-friendly programs, practices, and partnerships. This symposium will feature AFU advocates discussing innovate ways in which older adults can serve as teaching allies and support the educational mission of higher education. Farah (Lasell University) will discuss how older adults can engage in diverse teaching and learning activities with examples as crime scenario developers in a forensics class, conversation partners in an international oral communication class, and professional interviewers in an internship skills class. Kaye (University of Maine) will discuss how older adults can serve as citizen scientists performing critical functions in participatory research and in community-based test-beds and co-design laboratories. Ermer (Montclair State University) will discuss how older adults can engage students in discourse as guest speakers and panel participants in classes across the curriculum. Manoogian (Western Oregon University) will describe the innovative ways that older adult students (for credit or audit) mentor and engage younger student peers in course activities as well as increase their own understanding of the aging process.

